# Heart rate variability analysis using electrocardiograms during cardio-ankle vascular index measurement shows good agreement with resting electrocardiogram-based analysis in patients with diabetes: a retrospective cross-sectional study

**DOI:** 10.1007/s13340-025-00869-z

**Published:** 2026-01-21

**Authors:** Yuka Shibata, Toshiki Kiyose, Tatsuhito Himeno, Masahiro Shimoda, Hirohiko Ando, Ayako Ito, Atsuo Itani, Toru Shimizu, Rion Miura, Mika Matsuoka, Kento Tsuzuki, Takahiro Shinozaki, Mikio Motegi, Tomohide Hayami, Hiromi Nakai-Shimoda, Makoto Kato, Emiri Miura-Yura, Takayuki Nakayama, Yoshiaki Morishita, Masaki Kondo, Shin Tsunekawa, Jiro Nakamura, Tetsuya Amano, Hideki Kamiya

**Affiliations:** 1 Division of Diabetes, Department of Internal Medicine, Aichi Medical University School of Medicine, 1-1 Yazakokarimata, Nagakute, Aichi 480-1195 Japan; 2 Department of Clinical Laboratory, Aichi Medical University Hospital, 1-1 Yazakokarimata, Nagakute, Aichi 480-1195 Japan; 3 Division of Cardiology, Department of Internal Medicine, Aichi Medical University School of Medicine, 1-1 Yazakokarimata, Nagakute, Aichi 480-1195 Japan; 4 Department of Diabetes, Endocrinology and Metabolism/Department of Rheumatology and Clinical Immunology, Gifu University Graduate School of Medicine, 1-1 Yanagido, Gifu, 501-1194 Japan; 5 TDE Healthcare Corporation, TOSAKI Meito Clinic for Diabetes and Endocrinology, 7-32 Iwasaki-cho, Nisshin, Aichi 470-0125 Japan

**Keywords:** Diabetic autonomic neuropathy, Cardiovascular autonomic neuropathy, Heart rate variability, Power spectral analysis

## Abstract

**Background:**

Cardiovascular autonomic neuropathy (CAN) is a common but underrecognized diabetic complication. Although heart rate variability (HRV) analysis is a less invasive alternative to cardiovascular autonomic reflex tests, it is not routinely performed due to time and equipment constraints. This study aimed to assess whether HRV analysis using electrocardiograms (ECG) recorded during cardio-ankle vascular index (CAVI) measurement can serve as a practical substitute for conventional resting ECG-based HRV assessment.

**Methods and results:**

This cross-sectional study enrolled 48 patients with diabetes and 20 healthy controls. HRV spectral indices were calculated from ECG recorded during both resting (3-min HRV) and CAVI measurement (CAVI-HRV). Agreement between HRV indices obtained under different conditions was evaluated by intraclass correlation coefficients and Bland–Altman analyses. Correlations between HRV parameters and clinical indices were examined. Participants with diabetes showed significantly lower HRV (especially high-frequency power), and reduced coefficient of variation of RR intervals. HF power of CAVI-HRV showed good agreement with 3-min HRV, whereas LF power showed only moderate concordance. HRV spectral parameters did not significantly correlate with severity of sensorimotor polyneuropathy.

**Conclusions:**

HRV analysis performed during CAVI measurement reliably assesses parasympathetic function in diabetes. This approach may provide a convenient, accessible strategy for early CAN screening.

## Introduction

Among the many complications arising from diabetes, cardiovascular autonomic neuropathy (CAN) is one of the most common, yet remains substantially underrecognized and undertreated [1, 2]. A meta-analysis reported that CAN is associated with a relative risk of mortality of 3.45 (95% confidence interval: 2.66–4.47) indicating its significant impact on the prognosis of patients with diabetes [3].

Early detection of CAN is crucial for initiating timely interventions that may prevent further progression and improve both prognosis and quality of life [4]. However, the early manifestations of diabetic autonomic neuropathy are often vague or nonspecific—such as resting tachycardia, orthostatic symptoms, gastrointestinal dysmotility, and sexual dysfunction—and are frequently overlooked until the disease has advanced [2].

Traditionally, CAN is diagnosed using cardiovascular autonomic reflex tests (CARTs), which evaluate autonomic function through maneuvers such as deep breathing, postural changes, and the Valsalva maneuver [2]. While widely employed, these tests can transiently increase intrathoracic, intraocular, and intracranial pressures, posing potential risks, particularly in patients with diabetic retinopathy.

Furthermore, CARTs are time-consuming and resource-intensive, making it impractical to apply them universally to all patients with diabetes. As a result, there is growing demand for simpler and safer screening methods. Recently, heart rate variability (HRV) analysis has attracted attention as a potential alternative. HRV-based assessments are less invasive, easier to perform, and may offer comparable diagnostic utility [5–7]. Nevertheless, their clinical value remains under active investigation, and consensus regarding their role in CAN screening has not yet been reached.

In this context, the present study explores a novel approach to assessing HRV using electrocardiogram (ECG) recorded during measurement of the cardio-ankle vascular index (CAVI), a non-invasive indicator of arterial stiffness. Because CAVI is widely used to evaluate arteriosclerosis and is particularly relevant in patients with diabetes, combining HRV analysis with CAVI measurement may offer additional clinical value. Simultaneous assessment of autonomic function and arterial stiffness could enhance efficiency and broaden the applicability of routine screening in diabetes care. Specifically, we aim to investigate the correlation between HRV parameters derived from ECG obtained during CAVI measurement and those obtained from conventional resting ECG recordings. Our objective is to determine whether this method could provide a simpler, safer, and more accessible strategy for early CAN detection, potentially reducing dependence on CARTs and improving clinical outcomes in individuals with diabetes.

## Materials and methods

### Study design and patients

This was a cross-sectional study that included 20 healthy subjects and 48 patients with diabetes who were admitted to Aichi Medical University Hospital for glycemic control between April 2023 and March 2024. All participants provided written informed consent prior to inclusion. Patients receiving α-adrenergic or β-adrenergic blockers, those with persistent atrial fibrillation, and those with implanted pacemakers were excluded from the study. IRB information: The ethics committee of Aichi Medical University Hospital (NO. 2022-099).

### Clinical and neurological evaluation

All participants underwent clinical assessment, including documentation of age, sex, height, and body weight. Resting ECG and CAVI were carried out. In the diabetes group, additional vascular assessments were incorporated, including brachial-ankle pulse wave velocity (baPWV), toe-brachial index (TBI), ankle-brachial index (ABI), and intima-media thickness of the common carotid artery measured 10 mm distal to the carotid bulb (IMT-C10). Duration of diabetes was recorded based on patient interviews. Laboratory evaluations encompassed measurements of plasma glucose, HbA1c, triglycerides, total cholesterol, high-density lipoprotein cholesterol (HDL-C), low-density lipoprotein cholesterol (LDL-C), blood urea nitrogen, uric acid, and creatinine. Nerve conduction studies (NCS) were conducted in a temperature-controlled (20–27 °C), shielded environment. Assessments included motor and sensory conduction of the median nerves in the upper limbs and the tibial and sural nerves in the lower limbs. The severity of diabetic polyneuropathy was evaluated according to Baba’s differentiation classification, a five-stage system based primarily on nerve conduction study (NCS) results, especially sural sensory nerve action potential (SNAP) and tibial compound muscle action potential (CMAP) amplitudes [8].

In this system:

- Stage 0 indicates the absence of neuropathy, defined by entirely normal NCS findings.
- Stage 1 corresponds to early or mild neuropathy, characterized by the presence of any of the following: reduced tibial motor nerve conduction velocity (< 40 m/s), reduced sural sensory nerve conduction velocity (< 40 m/s), prolonged tibial minimal F-wave latency (exceeding {12.8 + 0.22 × Height in cm} ms).
- Stage 2 reflects moderate neuropathy and is defined by a reduction in sural SNAP amplitude to below 5 µV.
- Stage 3 represents more advanced neuropathy, with both sural SNAP amplitude < 5 µV and a tibial CMAP amplitude ranging between ≥ 2 and < 5 mV.

This classification enables a graded assessment of neuropathy severity based on objective electrophysiological parameters.

### HRV analysis

All procedures were conducted between 3:00 p.m. and 4:00 p.m. to minimize diurnal variation in autonomic function. After a clinical interview, two types of ECG recordings were acquired: a 3-minute resting ECG recorded in the supine position using the VaSera™ VS-2500 (Fukuda Denshi Co., Ltd., Tokyo, Japan) and a simultaneous ECG recorded for at least 2 min during CAVI measurement using the same device. CAVI measurement itself required approximately 5 min and was performed according to the manufacturer’s standard protocol.

HRV was analyzed from each ECG using frequency domain methods based on RR intervals. For the 3-minute and CAVI-associated recordings, a single continuous segment was analyzed. Additionally, the coefficient of variation of RR intervals (CV_R–R_) was calculated from the 3-minute ECG. Spectral power (ms^2^) was calculated for each frequency band using a Fast Fourier transform (FFT) algorithm. In the spectral analysis of HRV, 128 RR intervals from the 3-minute ECG and 64 RR intervals from the CAVI-ECG were applied. The low-frequency (LF) band was defined as 0.039–0.148 Hz and the high-frequency (HF) band as 0.148–0.398 Hz.

### Statistical analysis

All statistical analyses were performed using IBM SPSS Statistics version 29.0. Student’s t-test was used to compare groups. Intraclass correlation coefficients (ICCs) were calculated to assess the degree of agreement between HRV parameters measured under different conditions, in order to evaluate the consistency and interchangeability of the methods. ICCs were computed using a two-way mixed-effects model with absolute agreement for single measures. Additionally, Bland–Altman analysis was conducted to assess the agreement between the two methods. To explore the relationships between HRV spectral parameters and other clinical indices, correlation analyses were performed. Pearson’s correlation coefficient was used to assess linear associations. A *p*-value of less than 0.05 was considered statistically significant. The study protocol was approved by the Ethics Committee of Aichi Medical University (approval number 2022-099).

## Results

The clinical characteristics of patients with diabetes are shown in Table 1. This table includes demographic data, blood test results, and physiological parameters. According to Baba’s differentiation classification, more than half of the patients exhibited some degree of nerve conduction abnormality, suggesting a high prevalence of diabetic neuropathy among the study population (Table 1).

**Table 1:** Clinical characteristics of the study participants

Variable	Number	Value
Demographic and clinical characteristics		
Age (years)	48	58.3 ± 13.6
Sex (male/female)	48	31/17
Duration of diabetes (years)	43	9.2 ± 11.0
BMI (kg/m^2^)	48	25.7 ± 5.5
Laboratory data		
HbA1c (%)	47	9.2 ± 3.2
Fasting blood glucose (mg/dL)	46	149.3 ± 69.2
TC (mg/dL)	41	187.3 ± 45.2
LDL-C (mg/dL)	36	108.1 ± 38.5
HDL-C (mg/dL)	36	46.8 ± 13.4
TG (mg/dL)	37	164.0 ± 84.3
Serum creatinine (mg/dL)	47	1.09 ± 1.37
Urea nitrogen (mg/dL)	47	16.5 ± 12.1
eGFR (mL/min/1.73m^2^)	47	81.7 ± 37.8
Uric acid (mg/dL)	42	5.35 ± 1.68
ACR (mg/gCr)	33	97.8 ± 248.9
HRV		
LF, 3-min ECG (ms^2^)	48	273.7 ± 348.9
HF, 3-min ECG (ms^2^)	48	142.1 ± 228.9
LF/HF, 3-min ECG	48	2.88 ± 3.30
LF, CAVI-ECG (ms^2^)	46	267.1 ± 283.1
HF, CAVI-ECG (ms^2^)	46	169.9 ± 296.4
LF/HF, CAVI-ECG	46	3.23 ± 3.74
CV_R-R_ (%)	47	2.90 ± 1.44
Other physiological tests		
CAVI	48	8.45 ± 1.63
baPWV (m/s)	32	1621 ± 434.7
ABI	31	1.10 ± 0.30
TBI	30	0.77 ± 0.11
IMT-C10 (mm)	32	0.75 ± 0.11
QTc, Bazett (s)	33	0.428 ± 0.05
QTc, Fridericia (s)	33	0.406 ± 0.03
MCV, Median (m/s)	33	52.7 ± 4.9
CMAP, Median (mV)	33	14.3 ± 4.2
MCV, Tibial (m/s)	33	41.2 ± 5.9
Minimum F wave latency, Tibial (ms)	32	50.2 ± 5.8
CMAP, Tibial (mV)	33	18.9 ± 8.6
SCV, Median (m/s)	32	47.3 ± 7.0
SNAP, Median (µV)	32	30.5 ± 19.1
SCV, Sural (m/s)	32	45.9 ± 5.6
SNAP, Sural (µV)	32	10.7 ± 8.7
BDC (stage 0/1/2/3/4)	33	14/12/6/0/1

Values are presented as mean ± standard deviation

BMI: body mass index; HbA1c: glycated hemoglobin; TC: total cholesterol; LDL-C: low-density lipoprotein cholesterol; HDL-C: high-density lipoprotein cholesterol; TG: triglyceride; eGFR: estimated glomerular filtration ratio; ACR: albumin-to-creatinine ratio; HRV: heart rate variability; LF: low-frequency component of HRV; HF: high-frequency component of HRV; LF/HF: ratio of low- to high-frequency power; 3-min ECG: electrocardiography recorded for 3 min at rest; CAVI-ECG: electrocardiography recorded during cardio-ankle vascular index measurement; CV_R–R_: coefficient of variation of R–R intervals; CAVI: cardio-ankle vascular index; baPWV: brachial-ankle pulse wave velocity; ABI: ankle-brachial index; TBI: toe-brachial index; IMT-C10: intima-media thickness at the common carotid artery 10 mm from the bifurcation; QTc, Bazett: corrected QT interval using Bazett’s formula; QTc, Fridericia: corrected QT interval using Fridericia’s formula; MCV: motor nerve conduction velocity; CMAP: compound muscle action potential amplitude; SCV: sensory nerve conduction velocity; SNAP: sensory nerve action potential amplitude; Median: median nerve; Tibial: tibial nerve; Sural: sural nerve; BDC: Baba’s Differentiation Classification (stage 0 to 4)

A comparison between patients with diabetes and non-diabetic individuals is presented in Table 2. Patients with diabetes had significantly higher BMI and CAVI. HF power derived from ECG during CAVI measurement (CAVI-ECG) was significantly lower in the diabetic group. LF and HF power obtained by other measurement methods also tended to be lower. Additionally, the CV_R-R_ was significantly reduced in the diabetic group (Table 2).

**Table 2:** Comparison of autonomic nervous function parameters between participants with or without diabetes

Variable	Non-diabetes (*n* = 20)	Diabetes (*n* = 48)	*p*-value
Age (years)	51.4 ± 16.5	58.3 ± 13.6	0.074
BMI (kg/m^2^)	22.5 ± 3.6	25.7 ± 5.5	0.017*
SBP (mmHg)	120.0 ± 14.6	128.1 ± 14.4	0.039*
DBP (mmHg)	80.6 ± 11.5	82.5 ± 8.9	0.532
HR (/min)	68.4 ± 9.7	73.5 ± 13.4	0.071
LF, 3-min ECG (ms^2^)	253.8 ± 244.6	273.7 ± 348.9	0.176
HF, 3-min ECG (ms^2^)	203.6 ± 218.8	142.1 ± 228.9	0.114
LF/HF, 3-min ECG	2.65 ± 2.75	2.88 ± 3.30	0.787
LF, CAVI-ECG (ms^2^)	383.3 ± 416.3	267.1 ± 283.1	0.126
HF, CAVI-ECG (ms^2^)	344.3 ± 428.6	169.9 ± 296.4	0.021*
LF/HF, CAVI-ECG	2.01 ± 1.54	3.23 ± 3.74	0.187
CV_R–R_ (%)	3.68 ± 1.34	2.90 ± 1.44	0.024*
CAVI	7.65 ± 1.34	8.45 ± 1.63	0.031*

BMI: body mass index; SBP: systolic blood pressure; DBP: diastolic blood pressure; HR: heart rate; LF: low-frequency power; HF: high-frequency power; LF/HF: ratio of low- to high-frequency power; 3-min ECG: values obtained from 3-minute electrocardiogram recording at rest; CAVI-ECG: values obtained from electrocardiogram recording during cardio-ankle vascular index measurement; CV_R−R_: coefficient of variation of R-R intervals; CAVI: cardio-ankle vascular index

**Fig. 1:**
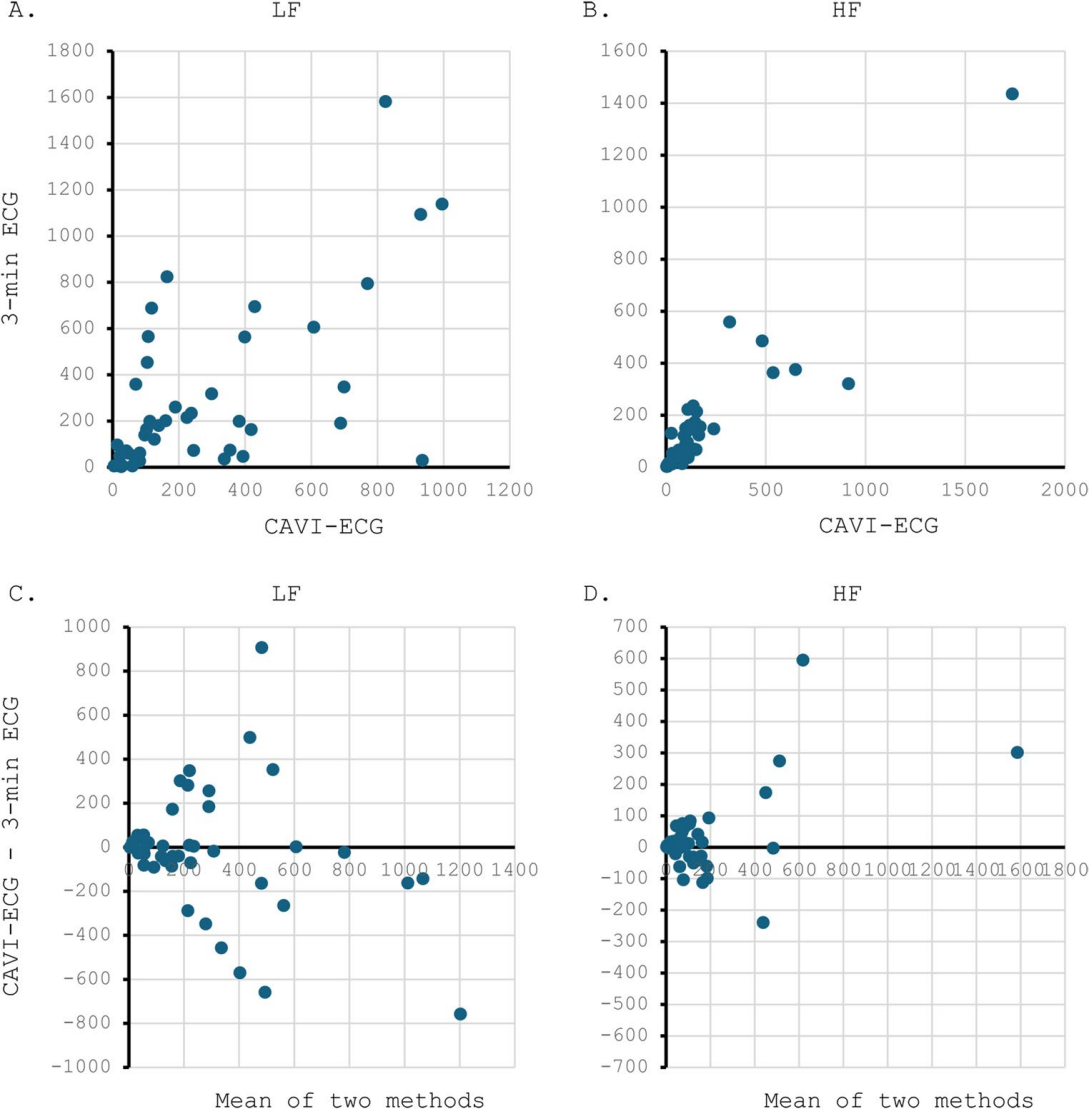
Visualization of heart rate variability parameters derived from 3-min ECG and CAVI-ECG. Panel A illustrates the scatter plot of low-frequency power obtained from the two methods, and panel B shows the corresponding plot for high-frequency power. Panels C and D present the Bland–Altman plots for low-frequency and high-frequency power, respectively

ICC was calculated to assess the agreement between HRV derived from a 3-minute resting ECG (3-min ECG HRV) and from CAVI-ECG (CAVI-ECG HRV) for LF and HF. For LF, the agreement was moderate in participants with diabetes and poor in healthy controls; when both groups were combined, it remained moderate. In contrast, HF showed good agreement in both groups as well as in the overall sample (Table 3). Scatter plots and Bland–Altman plots based on data from participants with diabetes are presented in Fig. 1. Greater values tended to be associated with larger variability.

**Table 3:** Agreement between two heart rate variability measurement methods assessed by intraclass correlation coefficient

	CAVI-ECG	3-min ECG	ICC
DM			
LF	267.1 ± 283.1	273.7 ± 348.9	0.617 (0.400, 0.768)
HF	169.9 ± 296.4	142.1 ± 228.9	0.897 (0.821, 0.941)
Non-DM			
LF	383.3 ± 416.3	253.8 ± 244.6	0.293 (− 0.187, 0.661)
HF	344.3 ± 428.6	203.6 ± 218.8	0.723 (0.399, 0.887)
Total			
LF	299.8 ± 326.8	267.9 ± 320.1	0.505 (0.298, 0.667)
HF	219.0 ± 344.3	160.2 ± 226.2	0.823 (0.725, 0.889)

DM: patients with diabetes, non-DM: non-diabetic healthy participants, Total: DM and non-DM, LF: low-frequency power, HF: high-frequency power, Data of LF and HF are presented as mean ± standard deviation, ICC: intraclass correlation coefficient, Data of ICC are presented as ICC (95% confidence intervals)

Results of the correlation analysis between HRV spectral parameters and other clinical indices are presented in Table 4. LF and HF showed a weak negative correlation with age, while no significant correlations were found with glycemic indices. Among nephropathy markers, eGFR was weakly correlated with the LF component of 3-min ECG HRV. With respect to atherosclerosis-related indices, LF showed a weak correlation with baPWV, and the LF component calculated from the 3-min ECG recording was also weakly correlated with TBI. Regarding other autonomic function tests, CV_R–R_ showed a moderate correlation with both LF and HF. QTc (Bazett’s formula) was weakly correlated with LF and strongly correlated with HF. No significant correlations were observed between HRV parameters and NCS results.

**Table 4:** Correlation analysis between HRV indices and other clinical parameters

Variable		LF, 3-min ECG	HF, 3-min ECG	LF, CAVI-ECG	HF, CAVI-ECG
Age	Pearson’s *r*	**− 0.397**	**− 0.326**	− 0.278	− 0.274
	*p* value	**0.005**	**0.024**	0.062	0.065
BMI	Pearson’s *r*	− 0.285	− 0.258	− 0.056	− 0.269
	*p* value	0.050	0.077	0.712	0.071
HbA1c	Pearson’s *r*	0.028	− 0.079	0.056	− 0.001
	*p* value	0.898	0.712	0.794	0.997
Fasting blood glucose	Pearson’s *r*	− 0.041	− 0.073	− 0.123	− 0.028
	*p* value	0.787	0.627	0.427	0.859
eGFR	Pearson’s *r*	**0.433**	0.139	0.225	0.122
	*p* value	**0.002**	0.353	0.137	0.424
ACR	Pearson’s *r*	− 0.009	0.219	0.136	0.243
	*p* value	0.962	0.221	0.449	0.174
CAVI	Pearson’s *r*	− 0.071	− 0.106	− 0.137	− 0.056
	*p* value	0.634	0.475	0.364	0.709
baPWV	Pearson’s *r*	**− 0.455**	− 0.308	**− 0.458**	− 0.219
	*p* value	**0.009**	0.086	**0.010**	0.237
ABI	Pearson’s *r*	− 0.177	0.016	− 0.200	0.086
	*p* value	0.332	0.932	0.280	0.647
TBI	Pearson’s *r*	**0.384**	0.234	0.360	0.232
	*p* value	**0.036**	0.213	0.055	0.225
IMT-C10	Pearson’s *r*	− 0.268	− 0.231	− 0.353	-0.199
	*p* value	0.138	0.203	0.051	0.282
CV_R–R_	Pearson’s *r*	**0.713**	**0.554**	**0.502**	**0.532**
	*p* value	**<0.001**	**<0.001**	**<0.001**	**<0.001**
QTc, Bazett	Pearson’s *r*	**0.416**	**0.762**	**0.437**	**0.736**
	*p* value	**0.016**	**<0.001**	**0.012**	**<0.001**
QTc, Fridericia	Pearson’s *r*	0.118	− 0.108	0.081	− 0.069
	*p* value	0.513	0.549	0.659	0.707
MCV, Tibial	Pearson’s *r*	− 0.045	0.109	0.205	0.121
	*p* value	0.804	0.547	0.260	0.510
Minimum F wave latency, Tibial	Pearson’s *r*	0.222	− 0.002	− 0.101	− 0.010
	*p* value	0.229	0.990	0.595	0.960
CMAP, Tibial	Pearson’s *r*	0.186	0.344	0.115	0.334
	*p* value	0.299	0.050	0.531	0.062
SCV, Sural	Pearson’s *r*	− 0.087	-0.078	0.082	− 0.027
	*p* value	0.635	0.671	0.661	0.886
SNAP, Sural	Pearson’s *r*	− 0.112	− 0.070	− 0.140	− 0.118
	*p* value	0.543	0.704	0.453	0.527

BMI: body mass index; HbA1c: glycated hemoglobin; eGFR: estimated glomerular filtration ratio; ACR: albumin-to-creatinine ratio; HRV: heart rate variability; LF: low-frequency component of HRV; HF: high-frequency component of HRV; 3-min ECG: electrocardiography recorded for 3 min at rest; CAVI-ECG: electrocardiography recorded during cardio-ankle vascular index measurement; CV_R−R_: coefficient of variation of R-R intervals; CAVI: cardio-ankle vascular index; baPWV: brachial-ankle pulse wave velocity; ABI: ankle-brachial index; TBI: toe-brachial index; IMT-C10: intima-media thickness at the common carotid artery 10 mm from the bifurcation; QTc, Bazett: corrected QT interval using Bazett’s formula; QTc, Fridericia: corrected QT interval using Fridericia’s formula; MCV: motor nerve conduction velocity; CMAP: compound muscle action potential amplitude; SCV: sensory nerve conduction velocity; SNAP: sensory nerve action potential amplitude; Tibial: tibial nerve; Sural: sural nerve. Pearson’s *r* indicates Pearson’s correlation coefficient. Variables with *P* < 0.05 are shown in bold

## Discussion

In this study, we evaluated the potential of HRV analysis using ECG signals recorded during CAVI measurement as an alternative to conventional resting ECG, primarily for early detection of CAN in patients with diabetes. Our findings demonstrated good agreement in HF power between CAVI-ECG and resting 3-min ECG across all participant groups, indicating that parasympathetic activity can be robustly assessed within the context of CAVI measurement. This is particularly significant as previous research has often been limited by the need for separate, dedicated autonomic testing sessions, which may not be feasible in routine clinical practice. The ability to derive reliable autonomic markers from ECG obtained during standard vascular assessments could therefore streamline clinical workflows and improve early CAN detection.

In contrast, the moderate to poor reproducibility of LF power observed in diabetic and control groups respectively is consistent with previous reports describing the susceptibility of LF measures to physiological and technical variability [9]. This aligns with growing consensus that HF-related parameters may provide more specific and clinically useful indications of early autonomic dysfunction, particularly within the diabetic population. Notably, our observation of reduced HF and CV_R–R_ in patients with diabetes supports earlier meta-analytic work that HRV parameters are diminished even in the subclinical phase of CAN [10]. These results reinforce the clinical relevance of HRV as a potentially valuable screening tool and highlight the need to prioritize parasympathetic indices for early detection.

Our findings also point to potential broader applications in routine health checks among high-risk patients. Given the rising global burden of diabetes and the often-insidious progression of CAN, incorporating HRV measurement during CAVI testing could allow large-scale, non-invasive screening without significant increase in testing burden. However, several practical issues remain before this approach can be implemented more widely in clinical practice. For instance, methodological variability in HRV acquisition and analysis currently limits inter-study comparability and clinical reliability [11]. Moreover, factors such as medication use, comorbid conditions, and acute physiological states may influence HRV indices and should be considered in study design and clinical interpretation. Establishing population-specific reference values and standardized measurement conditions will be essential for broader adoption and for maximizing the clinical utility of HRV analysis.

Despite these strengths, there are notable limitations that must be considered. The relatively modest sample size and the absence of gold-standard CARTs for CAN restrict the generalizability of our findings and preclude meaningful assessment of diagnostic accuracy. Additionally, possible confounders such as respiratory rate during CAVI measurement were not controlled and could have influenced HRV parameters. Another limitation is that the number of RR intervals used for spectral analysis differed between the 3-min ECG and CAVI-ECG, which may have contributed to the variability observed in LF power. The potential influence of cuff pressurization during CAVI on autonomic indices should also be acknowledged. These issues underscore the need for future studies that incorporate larger, more diverse cohorts, concurrent gold-standard autonomic testing, and standardized measurement and analysis conditions.

It is also important to contextualize these findings in light of previous research indicating that the use of multiple autonomic indices yields higher predictive validity for mortality and major cardiovascular events than single-index approaches [3, 12]. For example, integrating ECG-derived HRV with other autonomic measures, such as the QTc, has been associated with improved risk prediction in diabetic populations [12]. This suggests that while CAVI-ECG derived HRV is a promising and accessible tool, it should ideally be implemented alongside other validated autonomic and electrophysiological indices. Building on recent recommendations, our future studies will prospectively assess the utility of combined HRV, QTc, CV_R–R_, and additional readily available indices to identify individuals at highest risk for adverse cardiovascular outcomes. Prospective validation, including tracking of clinical endpoints, will be critical for establishing the true prognostic value of these combined approaches.

Finally, while this study focused on diabetic participants, future research should expand these analyses to additional populations at risk for CAN, such as elderly individuals or those with metabolic syndrome, to further elucidate the generalizability of CAVI-ECG derived HRV assessment and to facilitate risk stratification on a broader scale. Establishing the incremental value, user-friendliness, and cost-effectiveness of this approach will be crucial for its meaningful integration into real-world practice.

In summary, our results suggest that ECG-derived HRV recorded during CAVI measurement represents a promising, accessible, and efficient method for evaluating autonomic function, particularly parasympathetic modulation, in diabetic patients. With further validation and refinement, this integrated approach could enhance early CAN detection and make comprehensive vascular and autonomic screening a feasible component of routine diabetes care.

## Data Availability

The data supporting the findings of this study are available from the corresponding author upon reasonable request.
